# Prevalence and factors associated with anxiety, depression and burnout in gynecology and obstetrics residents during the COVID-19 pandemic

**DOI:** 10.61622/rbgo/2024AO17

**Published:** 2024-03-15

**Authors:** Maria Luiza de Castro Amaral, Isabela Michel da Silva, Alexandre Ferreira Bello, Franciele Cascaes da Silva, Gustavo Salata Romão, Alberto Trapani

**Affiliations:** 1 University of Southern Santa Catarina Palhoça SC Brazil University of Southern Santa Catarina, Palhoça, SC, Brazil.; 2 Univesity of Ribeirão Preto Ribeirão Preto SP Brazil Univesity of Ribeirão Preto, Ribeirão Preto, SP, Brazil.; 3 Federal University of Santa Catarina Florianýpolis SC Brazil Federal University of Santa Catarina, Florianýpolis, SC, Brazil.

**Keywords:** Mental health, Medical residency, Internship and residency, Anxiety, Depression, Burnout, psychological, Gynecology, Obstetrics, COVID-19, Pandemics, Brazil

## Abstract

**Objective:**

To determine the prevalence of anxiety, depression and burnout in residents of Gynecology and Obstetrics during COVID-19 pandemic in Brazil and its associated factors.

**Methods:**

Cross-sectional study involving all regions of Brazil, through the application of a sociodemographic questionnaire, the Hospital Anxiety and Depression Scale (HAD) and the Maslach Burnout Inventory (MBI-HSS) instrument. Multivariate analysis was performed after adjusting the Poisson model.

**Results:**

Among the 719 participating medical residents, screening was positive for anxiety in 75.7% and for depression in 49.8% of cases. Burnout syndrome was evidenced in 41.3% of the physicians studied. Those with depression are more likely to have anxiety (OR 0.797; 95%CI 0.687 - 0.925) and burnout syndrome (OR 0.847 95%CI 0.74 - 0.97). Residents with anxiety (OR 0.805; 95%CI 0.699 - 0.928) and burnout (OR 0.841; 95%CI 0.734 - 0.963) are more likely to have depression.

**Conclusion:**

High prevalence of anxiety, depression and burnout were found in residents of Gynecology and Obstetrics in Brazil, in addition to important correlations between anxiety-depression and depression-burnout.

## Introduction

Medicine is a profession recognized for its high standards of demand.^(1)^ Since graduation, during specializations, sub-specializations and even at the end of training there is a great physical and emotional demand, which can lead to disorders in the field of mental health.^(2)^ During this training, medical residency is a stressful stage, due to the long working hours, sleep deprivation and the great responsibility for the lives of other people.^(3)^ Based on the above considerations, it is important to be aware of the advent of mental disorders, such as anxiety, depression and burnout in this group of professionals.

Generalized Anxiety Disorder (GAD) is characterized by the fifth edition of the Diagnostic and Statistical Manual of Mental Disorders (DSM-5) as excessive anxiety and worry about various events or activities.^(4)^ According to the World Health Organization (WHO), anxiety affects 3.6% of the world population and is related to three or more of the following symptoms: restlessness or a feeling of being on edge, fatigue, difficulty concentrating, muscle tension and sleep disturbance.^(5)^ Relevant levels of anxiety may be experienced by 20-30% of resident physicians.^(6)^ For the assessment of depression and anxiety, one of the most used instruments is the Hospital Anxiety and Depression Scale (HADS),^(7)^ which has already been applied to a wide range of situations.^(8)^

According to the WHO, depression is a disorder that affects more than 300 million people (4.4% of the world population), being the main cause of disability worldwide.^(5)^ Major Depressive Disorder (MDD) involves symptoms such as depressed mood, loss of interest and pleasure, and decreased energy, which can impair an individual's ability to function at work or cope with daily life.^(4,5)^ Studies suggest that depression has higher levels among resident physicians when compared to the general public.^(9,10)^ Consequently, depressed residents make six times more mistakes than non-depressed residents.^(11)^ In addition, residents affected by depressive symptoms have a higher risk of career dissatisfaction, a higher risk of burnout, and a lower ability to maintain a healthy personal and professional relationship.^(12)^

In this context of mental disorders, Burnout Syndrome (BS) in medical residents has received increasing attention in recent years, due to the significant prevalence among these professionals and the serious damage that it causes, such as the deterioration of the quality of care to the patient, self-reports of medical errors, mental disorders and higher rates of suicidal ideation.^(3,13,14)^ Maslach et al.,^(15)^ defined BS as a psychological syndrome of emotional exhaustion, depersonalization and reduced personal fulfillment induced by repeated exposure to stressors in the workplace.^(2,16)^ Today, the most used instrument to quantify and qualify burnout in research is the Maslach Burnout Inventory (MBI) questionnaire.^(13)^

Obstetrics Gynecology (OBGYN) is a training that deals with both specialties at the same time. These are clinical-surgical specialties, which constitute one of the four major areas of medicine. It involves training a variety of skills, which include urgent and emergency care, as well as long working hours. Studies show a high prevalence of problems related to the well-being of resident physicians, with SB and MDD rates of 51.2% and 32%, respectively.^(14,17)^

As Brazil has become one of the countries with the highest number of COVID-19 cases, numerous hospitals have been adapted to receive patients with severe cases of the new virus, leading in several institutions to a modification of activities in medical residency.^(18)^ This moment of the pandemic brought an alert about the mental health of health professionals. In an Italian study, 96% of OBGYN residents reported that COVID-19 had a negative psychological impact in terms of mood swings and more than half had some degree of anxiety.^(19)^ Likewise, the SARS-Cov-2 pandemic brought the subject of SB even more to the fore,^(13)^ being documented in this period, in health professionals, high rates of depression, anxiety, stress and other mental disorders.^(20)^

According to WHO data, mental disorders cost the world economy one trillion dollars a year. The first step in implementing mental health services and effective psychological intervention measures is to recognize the mental health status of at-risk groups, such as healthcare professionals during the COVID-19 pandemic.^(21)^ It is extremely important to study the prevalence of such diseases in health professionals so that basic interventions and cost reduction begin to be thought.

In order to have a better understanding of the mental health of OBGYN residents in Brazil, notably during this pandemic scenario, this study aimed to assess the prevalence and factors associated with signs of anxiety, depression and burnout in gynecology residents and obstetrics during the COVID-19 pandemic in Brazil.

## Methods

This is a cross-sectional descriptive study. Residents from the first to the third year of residency programs in OBGYN, from all Brazilian regions, registered with the National Commission for Medical Residency of the Federation of Gynecology and Obstetrics Associations of Brazil (FEBRASGO) and who answered the questionnaires were included. The study did not include unofficial resident physicians (volunteers) or non-scholarship interns. It was carried out between August and December 2021, with non-probabilistic convenience sampling. The study was carried out in the descending phase of the second wave of the covid pandemic, in a period when vaccines were already available.

Resident physicians were invited to participate in the study through email messages, sent in the form of a hidden list. The message contained a link that directed the participant to fill out the instrument online, through the Google Forms platform, a secure tool that allows questionnaires to be completed and applied virtually, assuring participants secrecy and confidentiality. After reading and agreeing with the free and informed consent form, the system released the self-response instrument, which was applied in a single moment, with only one response per participant being accepted. After completing the questionnaire, the participant could only send it if all the questions were filled in with only one alternative. The participant could end the completion at any time, without being identified or suffering any embarrassment. There was no face-to-face contact with the participants.

The Hospital Anxiety and Depression Scale (HADS), validated for Brazil, is composed of 14 items, 7 of which are aimed at the assessment of anxiety (HADS-A) and 7 for the assessment of depression (HADS-D).^(22,23)^ Each of the items is scored from zero to three, with a maximum score of 21 points for each scale. A score equal to or greater than 9 on each scale was considered positive screening for anxiety or depression.^(24)^

The MBI instrument, a version used for the health area (HSS) and validated in Brazil, is composed of 22 items, with 9 questions about emotional exhaustion, eight questions about professional fulfillment and five about depersonalization.^(25,26)^ Responses were on a Likert-type scale with seven options: "never", "once a year or less", "once a month or less", "a few times a month", "once a week", "a few times a week" and "every day"

BS was defined by the presence of the three affected domains, that is, a high level of emotional exhaustion (≥ 27), a high level of depersonalization (≥ 10) and a low level of professional fulfillment (≤ 33).^(1,27)^

The independent variables analyzed were: age, gender, type of undergraduate university, source of financial resources, year of residence, region of Brazil, marital status, children, smoking, regular physical activity, weekly working time, supervision at residency, use of psychoactive drugs and use of illicit drugs.

Data were tabulated using Windows Excel software. In order to verify and compare the rates of problems in the sample evaluated, the data related to the variables burnout, anxiety and depression were dichotomized considering the presence or absence of the indicators, having as reference the normative data of the instruments used. Subsequently, the data were exported to the SPSS 16.0 program (IBM Corp., Armonk, NY, USA). Qualitative variables were described using absolute and relative frequencies, while quantitative variables were described as means and standard deviations for carrying out the descriptive analysis. The chi-square test (χ^2^) was used to test the homogeneity of proportions. Variables with p-value < 0.20 were submitted to Poisson regression analysis. To confirm whether the data fit the Poisson regression model, the GOODNESS OF FIT test was evaluated: p>0.05 and the OMNIBUS test: p<0.05. The established significance level was p < 0.05.

The study was approved by the ethics committee in research with human beings under the substantiated opinion number 4.874.49, and complied with the ethical precepts of the National Health Council (NHC), Resolutions 466/2012.

## Results

Of the total of 3859 registered resident physicians, 719 answered the questionnaires (18.63%). According to the regions, the prevalence of response taking into account registered residents, was 32.1% in the North (162 registered residents), 14.7% in the Northeast (530 registered residents), in the Midwest 11.1% (341 registered residents), in the Southeast 17.1% (2232 registered residents) and in the South 28.6% (594 registered residents). The most prevalent general characteristics of the resident physicians studied were age 28 years or less (58.3%), attending programs in the Southeast region (53.0%), female sex (86.1%), without a spouse or partner (64.1%), without children (92.4%), need financial supplementation in addition to the residency grant (91.8%), coming from private universities (66.2%), similar distribution in terms of year of residency, non -smoker (95.6%), without regular physical activity (52.0%), claim that the supervision of the program is inadequate (59.5%), do not use psychoactive medication (64.1%), do not use illicit drugs (90.8%) and have a weekly workload of more than 60 hours (82.0%). Screening was positive for anxiety in 544 cases (75.7%) and for depression in 358 cases (49.8%). BS was evidenced in 297 cases (41.3%). The general variables and by regions are in table 1.

**Table 1:** Descriptive table among Brazilian regions

Variables		North n(%)	Northeast n(%)	Midwest n(%)	Southeast n(%)	South n(%)	Total n(%)
Total of cases		52(7,2)	78(10,9)	38(5,3)	381(53,0)	170(23,6)	719(100,0)
Age group (years)							
	≤ 28	23(44,2)	43(55,1)	23(60,5)	233(61,1)	97(57,1)	419(58,3)
	> 28	29(55,7)	35(44,9)	15(39,5)	148(38,9)	73(42,9)	300(41,7)
Sex							
	Female	48(92,3)	68(87,2)	31(81,6)	329(86,3)	143(84,1)	619(86,1)
	Male	4(7,7)	10(12,8)	7(18,4)	52(13,7)	27(15,9)	100(13,9)
Married or stable union							
	Yes	19(36,5)	32(41,0)	21(55,3)	122(32,0)	64(37,6)	258(35,9)
	No	33(63,5)	46(59,0)	17(44,7)	259(68,0)	106(62,4)	461(64,1)
Kids							
	Yes	10(19,2)	12(15,4)	6(15,8)	17(4,5)	10(5,9)	55(7,6)
	No	42(80,8)	66(84,6)	32(84,2)	364(95,5)	160(94,1)	664(92,4)
Source of income							
	Just scholarship	3(5,8)	6(7,7)	3(7,9)	30(7,9)	17(10,0)	59(8,2)
	Scholarship + personal resources	29(55,8)	43(55,1)	17(44,7)	202(53,0)	66(38,8)	357(49,6)
	Scholarship + third-party support	20(38,4)	29(37,2)	18(47,4)	149(39,1)	87(51,2)	303(42,2)
Undergraduate University							
	Public	19(36,5)	27(34,6)	16(42,1)	134(35,2)	47(27,6)	243(33,8)
	Private	33(63,5)	51(65,4)	22(57,9)	247(64,8)	123(72,4)	476(66,2)
Year of residency							
	R1	23(44,2)	35(44,9)	15(39,5)	113(29,7)	63(37,1)	249(34,6)
	R2	17(32,7)	35(32,0)	14(36,8)	136(35,7)	50(29,4)	242(33,7)
	R3	12(23,1)	18(23,1)	9(23,7)	132(34,6)	57(33,5)	228(31,7)
Smoker							
	Yes	2(3,8)	2(2,6)	2(5,3)	17(4,5)	9(5,3)	32(4,4)
	No	50(96,2)	76(97,4)	36(94,7)	364(95,5)	161(94,7)	687(95,6)
Physical Activity							
	Yes	23(44,2)	38(48,7)	19(50,0)	185(48,6)	80(47,1)	345(48,0)
	No	29(55,8)	40(51,3)	19(50,0)	196(51,4)	90(52,9)	374(52,0)
Proper supervision							
	Yes	17(32,7)	36(46,2)	17(44,7)	144(37,8)	77(45,3)	291(40,5)
	No	35(67,3)	42(53,8)	21(55,3)	237(62,2)	93(54,7)	428(59,5)
Use of psychoactive medication							
	Yes	24(46,2)	13(16,7)	11(29,0)	141(37,0)	69(40,6)	258(35,9)
	No	28(53,8)	65(83,3)	27(71,0)	240(63,0)	101(59,4)	461(64,1)
Use of illicit drugs							
	Yes	1(1,9)	6(7,7)	4(10,5)	41(10,8)	14(8,2)	66(9,2)
	No	51(98,1)	72(92,3)	34(89,5)	340(89,2)	156(91,8)	653(90,8)
Working hours							
	> 60 hours/week	47(90,4)	59(75,6)	24(63,2)	315(82,7)	145(85,3)	590(82,0)
	≤ 60 hours/week	5(9,6)	19(24,4)	14(36,8)	66(17,3)	25(14,7)	129(18,0)
Anxiety							
	Yes	45(86,5)	49(62,8)	28(73,7)	302(79,3)	120(70,6)	544(75,7)
	No	7(13,5)	29(37,2)	10(26,3)	79(20,7)	50(29,4)	175(24,3)
Depression							
	Yes	30(57,7)	33(42,3)	17(44,7)	204(53,5)	74(43,5)	358(49,8)
	No	22(42,3)	45(57,7)	21(55,3)	177(46,5)	96(56,5)	361(50,2)
Burnout							
	Yes	23(44,2)	22(28,2)	17(44,7)	174(45,7)	61(35,9)	297(41,3)
	No	29(55,8)	56(71,8)	21(55,3)	207(54,3)	109(64,1)	422(58,7)

CW - Mid-West; R1 - first-year resident physician; R2 - second-year resident physician; R3 - third-year resident physician

Tables 2, 3 and 4 show the distribution of the variables studied according to the presence or absence of anxiety, depression and burnout.

**Table 2:** Association of variables with the presence or absence of anxiety

Variables		Anxiety		PR	CI	p-value
		Yes n(%)	No n(%)			
Age > 28 years		227(75,7)	73(24,3)	1,000	0,92 – 1,09	0,997
Female gender		485(78,4)	134(21,6)	1,328	1,12 – 1,57	< 0,0001
Married or stable union		205(79,5)	53(20,5)	1,081	0,99 – 1,17	0,076
Not having kids		503(75,8)	161(24,2)	1,067	0,57 – 2,01	0,841
Source of income						
	Just scholarship	46(78,0)	13(22,0)	1,033	0,90 – 1,19	0,667
	Scholarship + personal resources	263(73,7)	94(26,3)	0,949	0,87 – 1,03	0,217
	Scholarship + third-party support	235(77,6)	68(22,4)	1,044	0,96 – 1,13	0,312
	Public university graduation	185(76,1)	58(23,9)	1,009	0,92 – 1,10	0,833
Year of residency						
	R1	201(80,7)	48(19,3)	1,106	1,02 – 1,20	0,021
	R2	186(76,9)	56(23,1)	1,024	0,94 – 1,12	0,059
	R3	157(68,9)	71(31,1)	0,874	0,79 – 0,96	0,004
Non smoker		524(76,3)	163(23,7)	1,220	0,93 – 1,60	0,076
No regular physical activity		295(78,9)	79(21,1)	1,093	1,00 – 1,19	0,036
Inadequate supervision		351(82,0)	77(18,0)	1,237	1,13 – 1,36	< 0,0001
Use of psychoactive medication		229(88,8)	29(11,2)	1,299	1,20 – 1,40	< 0,0001
Use of illicit drugs		48(72,7)	18(27,3)	0,957	0,82 – 1,12	0,560
Working hours > 60 hours/week		460(78,0)	130(22,0)	1,896	1,26 – 2,86	0,002
Region						
	North	45(86,5)	7(13,5)	1,157	1,03 – 1,30	0,058
	Northeast	49(62,8)	29(37,2)	0,813	0,68 – 0,97	0,005
	Mid-west	28(73,7)	10(26,3)	0,972	0,80 – 1,18	0,770
	Southeast	302(79,3)	79(20,7)	1,107	1,02 – 1,20	0,017
	South	120(70,6)	50(29,4)	0,914	0,82 – 1,02	0,078
Depression		338(94,4)	20(5,6)	1,655	1,51 – 1,82	< 0,0001
Burnout		272(91,6)	25(8,4)	1,421	1,31 – 1,54	< 0,0001

PR - prevalence ratio; CI confidence interval; R1 - first-year resident physician; R2 - second-year resident physician; R3 - third-year resident physician

**Table 3:** Association of variables with the presence or absence of depression

Variables		Depression		PR	CI	p-value
		Yes n(%)	No n(%)			
Age > 28 years		160(53,3)	140(46,7)	1,129	0,97 – 1,31	0,108
Female gender		313(50,6)	306(49,4)	1,124	0,89 – 1,41	0,302
Married or stable union		140(54,3)	118(45,7)	1,148	0,99 – 1,33	0,073
Not having kids		328(49,4)	336(50,6)	0,898	0,67 – 1,21	0,463
Source of income						
	Just scholarship	31(34,4)	59(65,6)	1,040	0,77 – 1,40	0,800
	Scholarship + personal resources	181(33,6)	357(66,4)	1,024	0,86 – 1,21	0,779
	Scholarship + third-party support	146(32,5)	303(67,5)	0,963	0,81 – 1,14	0,670
	Public university graduation	121(49,8)	122(50,2)	1,000	0,73 – 1,36	0,999
Year of residency						
	R1	125(50,2)	124(49,8)	1,013	0,87 – 1,18	0,873
	R2	126(52,1)	116(47,9)	1,070	0,92 – 1,25	0,385
	R3	107(46,9)	121(53,1)	0,918	0,78 – 1,08	0,296
Non smoker		342(49,8)	345(50,2)	0,996	0,70 – 1,42	0,981
No regular physical activity		217(58,0)	157(42,0)	1,420	1,22 – 1,65	< 0,0001
Inadequate supervision		249(58,2)	179(41,8)	1,553	1,31 – 1,84	< 0,0001
Use of psychoactive medication		166(64,3)	92(35,7)	1,545	1,34 – 1,78	< 0,0001
Use of illicit drugs		29(43,9)	37(56,1)	0,872	0,66 – 1,16	0,318
Working hours > 60 hours/week		307(52,0)	283(48,0)	1,316	1,05 – 1,65	0,010
Region						
	North	30(57,7)	22(42,3)	1,081	0,85 – 1,38	0,546
	Northeast	33(42,3)	45(57,7)	0,765	0,59 – 1,00	0,029
	Mid-west	17(44,7)	21(55,3)	0,893	0,62 – 1,28	0,522
	Southeast	204(53,5)	177(46,5)	1,175	0,01 – 1,36	0,033
	South	74(43,5)	96(56,5)	0,841	0,70 – 1,02	0,062
	Anxiety	338(62,1)	206(37,9)	5,437	7,74 – 8,25	< 0,0001
Burnout		216(72,7)	81(27,3)	2,161	1,86 – 2,51	< 0,0001

PR - prevalence ratio; CI confidence interval; R1 - first-year resident physician; R2 - second-year resident physician; R3 - third-year resident physician

**Table 4:** Association of variables with the presence or absence of burnout

Variables		Burnout		PR	CI	p-value
		Yes n(%)	No n(%)			
Age > 28 years		117(39,0)	183(61,0)	0,935	0,83 – 1,06	0,288
Female gender		257(41,5)	362(58,5)	1,038	0,80 – 1,34	0,775
Married or stable union		105(40,7)	153(59,3)	0,977	0,81 – 1,17	0,804
Not having kids		279(42,0)	385(58,0)	1,284	0,87 – 1,89	0,179
Source of income						
Just scholarship		29(49,2)	30(50,8)	1,210	0,92 – 1,59	0,201
Scholarship + personal resources		154(43,1)	203(56,9)	1,092	0,92 – 1,30	0,322
Scholarship + third-party support		114(37,6)	189(62,4)	0,855	0,71 – 1,03	0,087
Public university graduation		101(41,6)	142(58,4)	1,009	0,84 – 1,21	0,921
Year of residency						
	R1	110(44,2)	139(55,8)	1,110	0,93 – 1,33	0,255
	R2	109(45,0)	133(55,0)	1,143	0,96 – 1,37	0,148
	R3	78(34,2)	150(65,8)	0,767	0,62 – 0,94	0,008
Non smoker		284(41,3)	403(58,7)	1,018	0,66 – 1,56	0,936
No regular physical activity		180(48,1)	194(51,9)	1,419	1,18 – 1,70	< 0,0001
Inadequate supervision		215(50,2)	213(49,8)	1,783	1,45 – 2,19	< 0,0001
Use of psychoactive medication		135(52,3)	123(47,7)	1,489	1,26 – 1,76	< 0,0001
Use of illicit drugs		28(42,4)	38(57,6)	1,030	0,77 – 1,38	0,847
Working hours > 60 hours/week		258(43,7)	332(56,3)	1,446	1,10 – 1,91	0,005
Region						
	North	23(44,2)	29(55,8)	1,077	0,78 – 1,48	0,657
	Northeast	22(28,2)	56(71,8)	0,657	0,46 – 0,95	0,013
	Mid-west	17(44,7)	21(55,3)	1,088	0,76 – 1,57	0,184
	Southeast	174(45,7)	207(54,3)	1,255	1,05 – 1,50	0,012
	South	61(35,9)	109(64,1)	0,835	0,67 – 1,50	0,100
	Anxiety	272(50,0)	272(50,0)	3,500	2,41 – 5,08	< 0,0001
Depression		216(60,3)	142(39,7)	2,689	2,18 – 3,32	< 0,0001

PR- prevalence ratio; CI confidence interval; R1 - first-year resident physician; R2 - second-year resident physician; R3 - third-year resident physician

With multivariate regression analysis (Table 5), it was found that resident physicians with probable depression have a higher risk of having positive screening for anxiety and BS. Those with signs of anxiety had a higher risk of positive screening for depression. Those with BS have a higher risk of positive screening for depression. All other variables were not associated with anxiety, depression or burnout.

**Table 5:** Multivariate regression

Variable		OR	CI	adjusted p
Anxiety				
	Female gender	0,901	0,75 – 1,08	0,264
	Married or stable union	0,956	0,83 – 1,10	0,535
	R1	0,918	0,78 – 1,08	0,314
	R2	0,964	0,82 – 1,13	0,655
	R3	0,991	0,88 – 1,22	0,891
	Non smoker	0,911	0,67 – 1,24	0,550
	No regular physical activity	1,021	0,89 – 1,17	0,765
	Inadequate supervision	0,960	0,84 – 1,10	0,563
	Use of psychoactive medication	0,912	0,79 – 1,06	0,223
	Work hours > 60 hours/week	0,958	0,81 – 1,13	0,620
	North region	0,956	0,65 – 1,41	0,821
	Northeast region	1,060	0,75 – 1,49	0,741
	Southeast region	0,978	0,98 – 0,72	0,883
	South region	1,019	0,74 – 1,40	0,907
	Depression	0,797	0,69 – 0,92	0,003
	Burnout	0,908	0,78 – 1,06	0,215
Depression				
	Age > 28 years	1,045	0,92 – 1,18	0,496
	Married or stable union	0,985	0,86 – 1,12	0,819
	No regular physical activity	0,944	0,83 – 1,07	0,361
	Inadequate supervision	0,955	0,84 – 1,08	0,467
	Use of psychoactive medication	0,928	0,81 – 1,06	0,273
	Work hours > 60 hours/week	0,978	0,84 – 1,14	0,779
	Northeast region	0,972	0,76 – 1,25	0,823
	Southeast region	0,979	0,81 – 1,18	0,825
	South region	1,014	0,82 – 1,25	0,894
	Anxiety	0,805	0,70 – 0,93	0,003
	Burnout	0,841	0,73 – 0,96	0,013
Burnout				
	Not having kids	1,062	0,85 – 1,32	0,587
	Depending on third-party resources	1,053	0,93 – 1,19	0,405
	R2	0,991	0,86 – 1,14	0,898
	R3	1,052	0,91 – 1,22	0,503
	No regular physical activity	0,952	0,84 – 1,07	0,426
	Inadequate supervision	0,928	0,84 – 1,05	0,227
	Use of psychoactive medication	0,960	0,84 – 1,09	0,536
	Work hours > 60 hours/week	0,967	0,83 – 1,12	0,663
	Northeast region	1,025	0,77 – 1,36	0,864
	Mid-west region	0,934	0,66 – 1,31	0,693
	Southeast region	0,969	0,77 – 1,22	0,793
	South region	0,996	0,79 – 1,28	0,975
	Anxiety	0,914	0,78 – 1,06	0,234
	Depression	0,847	0,74 – 0,97	0,016

OR - odds ratio; CI confidence interval; R1 - first-year resident physician; R2 - second-year resident physician; R3 - third-year resident physician

## Discussion

In the present study, we evaluated the prevalence of anxiety, depression and burnout among gynecology and obstetrics residents in Brazil and their possible associations with the predictor variables. In the population studied, anxiety was more prevalent (75.7%) when compared to depression and burnout, 49.8% and 41.3% respectively. This higher prevalence of anxiety compared to the other two was also found in a study of residents and medical students in Nepal.^(2)^ It is important to emphasize that the study in Nepal was carried out before the pandemic.

The prevalence of anxiety and depression is consistent with a study carried out with medical residents in Tunisia using the HAD scale, in which 74.1% had anxiety and 62% had depression.^(28)^ However, lower percentage are found in another study carried out in Mexico during COVID-19 pandemic, in which 10% of OBGYN residents had anxiety and 50% depression,^(29)^ such disparity in the anxiety can be explained by the difference in the instruments used. Estimates of the prevalence of depressive symptoms in medical residents average 28.8% according to a pre pandemic meta-analysis of 54 studies.^(10)^

Regarding the prevalence found for BS of 41.3%, defined as the presence of abnormal scores in all 3 domains (EE, DP, PA), it is in line with findings in the literature: in an analysis of 20 studies, the prevalence average burnout found in medical residents of all specialties was 35.1%, whereas the group that included OBGYN showed an even higher prevalence of 42.5%, which may suggest that residency in OBGYN is a factor higher risk for BS than other specialties. As in the present study, this analysis also used the MBI for diagnosis, however the syndrome was defined with the presence of only 1 dimension, which tends to increase the prevalence of the results.^(25)^

On the other hand, another study carried out with resident physicians in a hospital in Brazil, in which the presence of all three dimensions of the syndrome was also necessary as a diagnostic criterion for burnout, showed a prevalence of 27.9% of BS, lower than the one found in this article.^(27)^ One explanation for that is due to the fact that the study was carried out pre pandemic period.

Some possible explanations for the higher prevalence of anxiety, depression and burnout found in this study could be raised: different instruments used to measure outcomes, research with residents from different medical areas (not just OBGYN) and, above all, research carried out before the pandemic of COVID-19,^(27)^ which may suggest that after the beginning of the pandemic such outcomes became even more prevalent. In 2014, the prevalence of anxiety and depression in medical residents was 41.3% and 21.6%, respectively.^(30)^ Today, in the present study and in others carried out after the pandemic, these rates practically doubled, as in one carried out with doctors in China in which medical residents had a prevalence of 44.6% of anxiety, 50.4% of depression and 71.5% of stress^(31)^ and another carried out with residents in Brazil with rates of 40.3% for depression, 52.8% for anxiety and 48.6% for burnout.^(32)^ Such data support the hypothesis that the prevalence of mental illness increased after the COVID-19 pandemic.

Studies indicate that anxiety, depression and burnout are strongly correlated with each other. The presence of one predicts the occurrence of one of the other two or even both. Studies done in different settings found a significant association of burnout with depression and anxiety.^(11,33)^

The presence of anxiety confers a significantly greater risk for the development of other psychiatric disorders, such as depression, a finding reported by several authors and which reinforces what was found in the present study.^(11,34-36)^ This correlation between anxiety and depression can be understood because both situations need similar psychological conditions to manifest.^(35)^

The correlation between depression and the presence of burnout, demonstrated in the present study, is compatible with findings from other studies that also found significant associations between them.^(2,33,37)^ One explanation for this finding would be the proven predisposition to depression, reflected by family and personal history, associated with an increased risk of burnout.^(38)^

Some authors believe that depression follows burnout and that conditions of high psychological demands coupled with low decision-making power are significant predictors of subsequent depression.^(37,39,40)^ In addition, the stress caused by the accumulation of activities at home can cause both burnout and depression.^(37)^

The response rate (18.63%), the regional differences, use of scales to assess mental health and the characteristic of the self-administered questionnaires in a virtual way limit the conclusions of the study. It is important to point out that being a broad national study, involving several scenarios, some services had their routine more altered than others due to the COVID-19 pandemic. This heterogeneity limits some interpretations.

In addition, the lack of significance, after logistic regression, of some associations such as female sex, working hours and lack of physical activity, commonly found in similar studies, may be a consequence of the high prevalence of dependent variables during the studied period. Despite the lack of significance in the regression model, the correlation between lack of regular physical activity, inadequate supervision, use of psychoactive drugs and working hours with the dependent variables was highlighted. Also, qualitative studies or studies with mixed methods, with in-depth interviews or focus groups, can complement the findings and contribute with meta-inferences about the presented results.

It is worth mentioning that the scale for anxiety and depression is not an ideal instrument for diagnosis in the face of a structured interview, consisting of anamnesis and physical examination, being used for the purpose of stratifying the severity of conditions and also for purposes of defining the diagnosis in studies. The MBI is an instrument used for diagnosis in the doctor's office.

The large number of participants, including residents from all over the country, are strengths of the present study. The results against the correlation of the mental disorders found can serve to suggest strategies for changing the formatting of residency programs, thus aiming at improving the mental health of this group. In this context, some strategies can contribute to the prevention and mitigation of anxiety, depression and BS. Such strategies include limiting the resident physician's workload, qualified supervision of activities with formative and structured feedback, the implementation of mentoring programs and psycho-pedagogical support for apprentices.

## Conclusion

High prevalence of anxiety, depression and burnout (75.7%, 49.8% and 41.3% respectively) were found among residents of OBGYN in Brazil, in addition to important associations between anxiety-depression and depression-burnout. This study shows the need for interventions aimed at the mental health of resident doctors in OBGYN, with increased attention in times and aggravating situations such as the COVID-19 pandemic. Cultivating greater knowledge about anxiety, depression, burnout and their implications for medical residency is of strategic importance and can contribute to improving both workforce productivity and patient safety. Reiterating, despite not showing statistical significance, it is clear that it is necessary to be aware of the importance of performing regular physical activity, adequate supervision, non-use of psychoactive drugs and adequate working hours.
